# Antimicrobial Resistance Profiles of *Salmonella* Isolates on Chickens Processed and Retailed at Outlets of the Informal Market in Gauteng Province, South Africa

**DOI:** 10.3390/pathogens10030273

**Published:** 2021-03-01

**Authors:** Thelma M. Mokgophi, Nomakorinte Gcebe, Folorunso Fasina, Abiodun A. Adesiyun

**Affiliations:** 1 Department of Production Animal Studies, University of Pretoria, Private Bag X 04, Onderstepoort, Pretoria 0110, South Africa; mt.mokgophi@yahoo.com; 2 Agricultural Research Council–Bacteriology and Zoonotic Diseases Diagnostic Laboratory, Onderstepoort Veterinary Research, Private Bag X 05, Onderstepoort, Pretoria 0110, South Africa; GcebeN@arc.agric.za; 3 ECTAD, Food and Agriculture Organization of the UN, Dar es Salaam 14111, Tanzania & Department of Vet-erinary Tropical Diseases, University of Pretoria, Onderstepoort, Pretoria 0110, South Africa; folorunso.fasina@fao.org; 4 Department of Paraclinical Sciences, School of Veterinary Medicine, University of the West Indies, St. Augustine, Trinidad and Tobago

**Keywords:** *Salmonella* in chickens, antimicrobial resistance profiles, Gauteng province, chicken, South Africa

## Abstract

The study determined the antimicrobial resistance profiles of *Salmonella* on chickens processed and retailed at outlets of the informal markets in Gauteng province, South Africa. The study also investigated the relationship of antimicrobial resistant *Salmonella* to the source and type of samples and their serotypes. Carcass swabs, cloacal swabs and carcass drips were randomly collected from each of 151 slaughtered chickens from six townships. Isolation and identification were performed using standard and polymerase chain reaction (PCR) methods. The disc diffusion method was used to determine the resistance of *Salmonella* isolates to 16 antimicrobial agents and PCR to determine their serovars. Ninety-eight (64.9%) of the 151 chickens were contaminated with *Salmonella* of which 94.9% (93/98) were resistant serovars. The frequency of antimicrobial resistance of *Salmonella* isolates was high to erythromycin (94.9%) and spectinomycin (82.7%) but was low to ciprofloxacin (1.0%) and norfloxacin (1.0%) (*p* < 0.05). All 170 isolates of *Salmonella* tested exhibited resistance to one or more antimicrobial agents and the frequency varied significantly (*p* < 0.05) across the townships, the type of samples and the serovars. The prevalence of multidrug resistance (MDR) in *Salmonella* was 81.8% (139/170). Our findings pose zoonotic, food safety and therapeutic risks to workers and consumers of undercooked, contaminated chickens from these outlets.

## 1. Introduction

Globally, *Salmonella* spp. are known to cause high morbidity, mortality and economic losses in the poultry industry [1,2,3,4]. Additionally, *Salmonella* spp. are zoonotic agents with the potential to cause infection and disease in humans who consume improperly cooked contaminated poultry products [5,6,7,8]. In an effort to prevent, control or eradicate potential infection by *Salmonella* spp. and other pathogens in the poultry industry, antimicrobial agents are used for prophylaxis, therapy and as growth promoters [9,10,11,12,13]. In most developed countries, the use of antimicrobial agents is strictly controlled or banned for specific reasons in poultry through the enforcement of existing laws and regulations [5,6,14]. However, in most developing counties including South Africa, although laws exist to control the use of antimicrobial agents, there are challenges in their enforcement thereby resulting in their inappropriate use (overuse or abuse) [15,16]. In addition to the unregulated use of antimicrobial agents in South Africa, the Fertilizers, Farm Feed, Agricultural and Stock Remedies Act (Act 36, 1947) exists which legalizes the use of some antimicrobial agents, including tetracyclines, sulfonamides and trimethoprim. The Act also permits the purchase of over-the-counter antimicrobial agents without a prescription for use in the livestock industry [13,17,18]. This practice is likely to contribute to the development of resistant pathogens [13,19] or the occurrence of antimicrobial residues in animal tissues with public health implications [20,21].

In South Africa, over the years, outlets of the informal chicken market have emerged as popular venues for the population in the townships to access retailed chicken which is perceived to taste better than those from the commercial retail outlets. Furthermore, their prices are competitive and affordable, and they are conveniently located [22,23,24]. However, since these outlets are unregulated by health personnel and are considered illegal, concerns have been raised regarding their roadside locations, lack or inadequacy of potable water, and inadequate wastewater and solid waste disposal [22,25,26]. Sanitary practices have been reported to be poor, posing health risk to consumers of products from these outlets [22,23,27]. The chickens slaughtered at the informal market outlets usually originate from commercial poultry farms [23,28] and small poultry enterprises. Therefore, the findings on the chickens processed at the outlets may reflect the occurrence of antimicrobial resistance at the farms from where they originated. There is also the possibility of carcass contamination being affected by practices at the outlets which may cause cross-contamination of processed chickens.

The rising threat of antimicrobial resistance (AMR) has prompted the development of national action plans whose five strategic objectives include, among others, optimization of surveillance and early detection of AMR for reporting local, regional, and national resistance patterns to optimize empirical and targeted antibiotic choices [29,30]. 

There are a few reports of bacterial pathogens on chickens from the outlets of the informal market in Gauteng province, such as the prevalence and molecular characterization of *Staphylococcus aureus* isolates [22,31] and the isolation of *Salmonella* [32]. However, to date, there is no published documentation of the antimicrobial resistance profiles of *Salmonella* in chickens processed at outlets of the informal market in South Africa.

The current study determined the antimicrobial resistance profiles of *Salmonella* isolates recovered from chickens slaughtered, processed, and retailed at outlets of the informal market in Gauteng province. In addition, the study investigated the potential effect of township sources of chickens, types of samples processed and the serovars of isolates of *Salmonella* and their antimicrobial profiles. Another objective of the current study was to assess the potential effect of the practices at the outlets of the informal market on cross-contamination of carcass, cloacal swabs, and carcass drips by *Salmonella.* It is anticipated that the results from this study will contribute towards addressing AMR challenges in the country.

## 2. Results

### 2.1. Selection of Informal Market Outlets Used in the Current Study

A total of 151 chickens from which carcass swabs, cloacal swabs and carcass drips were each collected, originated from six townships (Atteridgeville, Garanguwa, Tembisa/Modise, Alexandra, Germiston and Soweto) across Gauteng province (Table 1).

### 2.2. Isolation, Identification and Confirmation of Salmonella

A total of 170 isolates of *Salmonella* that were recovered from chicken carcass swabs, cloacal swabs and carcass drips were identified and confirmed using conventional and PCR methods.

### 2.3. Determination of Salmonella Serovars by PCR and Conventional Serotyping

For the 170 isolates of *Salmonella* where the serovars were determined using both PCR and conventional slide agglutination test, a total of nine serovars were detected, namely, Bovismorbificans, Hadar, Dublin, Enteritidis, Mbandaka, Saintpaul, Thompson, Infantis, and Agona at varying frequencies. 

### 2.4. Selection of Antimicrobial Agents Used in the Study

Overall, the resistance of the 170 isolates of *Salmonella* to 16 antimicrobial agents that belonged to eight antimicrobial classes (Aminoglycosides, Beta lactams, Cephalosporins, Fluoroquinolones, Macrolides, Phenicols, Sulphonamides, and Tetracyclines) were determined and presented in Table 1.

### 2.5. Frequency of Chickens Contaminated with Salmonella

Of a total of 151 chickens (packed in individual bags) tested for the carriage of *Salmonella* in their carcass swabs, cloacal swabs and/or carcass drips, 98 (64.9%) were positive for the pathogen.

### 2.6. Detection of Resistance to Antimicrobial Agents According to the Townships, Types of Samples, and the Serovars of Salmonella

#### 2.6.1. Frequency of Detection of Resistant *Salmonella* in Chickens

The prevalence of antimicrobial resistant *Salmonella* in the 98 chickens positive for *Salmonella* was 94.6% (93/98) with the recovered isolates having exhibited resistance to one or more antimicrobial agents as shown in Table 1. For the six townships, antimicrobial resistance was highest to erythromycin, ranging from 60.0% (Ranguwa) to 100.0% (Atteridgeville, Tembisa/Modise and Soweto). Overall, for the 16 antimicrobial agents tested, the prevalence of resistance among *Salmonella* was high to erythromycin, 94.9% (93/98), spectinomycin, 82.7% (81/98) and streptomycin, 74.5% (73/98) but low to nalidixic acid, 1.0% (1/98), ciprofloxacin, 1.0% (1/98) and norfloxacin, 1.0% (1/98). The occurrence of resistant to one or more of the 16 antimicrobial agents within the townships was 13 (81.3%), 12 (75.0%), 9 (56.3%), 8 (50.0%), 1 (6.3%), and 1 (6.3%) for *Salmonella* isolates recovered from chickens sampled in outlets from Soweto, Germiston, Alexandra, Atteridgeville, Ranguwa, and Tembise/Modisa, respectively, and the differences were statistically significant (*p* = 0.0002). Overall, the frequency of resistance to antimicrobial agents in *Salmonella* isolates varied significantly (*p* < 0.001) between and within townships (Table 1).

#### 2.6.2. Frequency of Detection of Resistant *Salmonella* by Type of Sample Processed

Table 2 shows the frequency of resistance to antimicrobial agents among the isolates of *Salmonella* recovered from three types of samples (carcass, cloacal swabs and carcass drips). A total of 170 isolates of *Salmonella* comprising 54, 56 and 60 recovered from carcass swabs, cloacal swabs and carcass drips, respectively, were analyzed. All (100.0%) the *Salmonella* isolates exhibited resistance to one or more antimicrobial agent. Again, the frequency of resistance of *Salmonella* isolates from the three types of samples was high to erythromycin, 100.0% (170/170), spectinomycin, 88.8% (151/170) and streptomycin, 80.0% (136/170) but considerably low to ciprofloxacin, 0.6% (1/170), chloramphenicol, 1.8% (2/170) and norfloxacin, 1.8% (2/170). The differences were statistically significant (*p* < 0.05).

Across and within the three types of samples processed, the frequency of resistance to antimicrobial agents differed significantly (*p* < 0.001).

#### 2.6.3. Frequency of Detection of Resistant Strains among Serotypes of *Salmonella*

The frequency of antimicrobial resistant *Salmonella* among the serovars detected is displayed in Table 3. Of a total of 170 isolates tested, 103 (60.6%) were typable. The frequency of detection of the serovars among the 103 typable isolates of *S. enterica* was as follows: Bovismorbificans, 39.8%; Hadar, 17.5%; Dublin, 12.6%; Enteritidis, 10.7%; Mbandaka, 8.7%; Saintpaul, 5.8%; Thompson, 1.9%, Infantis, 1.9%, and Agona, 1.0%. The differences were statistically significant (*p* < 0.05). 

For the nine serovars of *Salmonella* detected, the frequency of resistance to one or more of the 16 antimicrobial agents varied considerably but a statistically significant difference was detected only for streptomycin (*p* < 0.029). All the typable isolates of the nine serovars were susceptible to nalidixic acid and ciprofloxacin.

For the 16 antimicrobial agents tested, the spectrum of resistance exhibited by isolates of *Salmonella* varied significantly across serovars, i.e., at least one isolate of each serovar exhibiting resistance to the different antimicrobial agents tested, and was high for Bovismorbificans, 75% (12/16), Enteritidis, 68.8% (11/16) and Hadar, 62.5% (10/16) but low at 31.3% (5/16) each for serovars Saintpaul, Thompson, Infantis, and Agona. The differences were statistically significant (*p* < 0.05). 

Within the nine serovars of *Salmonella* isolates tested, the prevalence of resistance to one or more of the 16 antimicrobial agents was detected for Bovismorficans (*p* < 0.01), Hadar (*p* < 0.01), Dublin (*p* < 0.01), Enteritidis (*p* < 0.01), Mbandaka (*p* < 0.01), and Saintpaul (*p* < 0.01).

#### 2.6.4. Prevalence of Resistance Patterns and Multi-resistant Isolates of *Salmonella*

A total of 13, 21 and 22 resistance patterns were detected in the isolates of *Salmonella* from carcass swabs, cloacal swabs and carcass drips, respectively. The same predominant resistance pattern, erythromycin-oxytetracycline-streptomycin-spectinmycin-doxycycline (E-OXT-S-SPE-DO) was detected in the three types of samples at a frequency of 42.6%, 41.1% and 43.3% of which 35 were multidrug resistant (MDR) (Table 4). Overall, the frequency of MDR among the *Salmonella* isolates was 81.8% (139/170) having exhibited resistance to three or more antimicrobial agents that belonged to eight antimicrobial classes tested. The occurrence of MDR in *Salmonella* isolates was 72.2% (39/54), 92.9% (52/56) and 80.0% (48/60) in carcass swabs, cloacal swabs and carcass drips, respectively. The differences were statistically significant (*p* = 0.0308). Of the 170 isolates of resistant *Salmonella*, 31 (18.2%) were resistant to antimicrobial agents that belonged to less than three antimicrobial classes (i.e., non-MDR). These were E-SPE (7, 4.1%), E (14, 8.2%), E-S-SPE (7, 4.1%), E-OXT-DO (2, 1.2%), and E-S (1, 0.6%). The frequency of MDR among the isolates of *Salmonella* was 81.8% (139/170). The number of antimicrobial agents in the patterns obtained for MDR isolates varied from 3 to 10 as follows: 3 antimicrobial agents (5,3.6%), 4 (23, 16.5), 5 (76, 54.7%), 6 (23, 16.5%), 7 (8, 5.8%), 8 (2, 1.4%), 9 (1, 0.7%), and 10 (1, 0.7%). Among MDR isolates, the predominant resistance pattern exhibited by *Salmonella* isolates from the three types of samples was E-OXT-S-SPE-DO, 51.8% (72/139).

## 3. Discussion

Our study demonstrated, for the first time in South Africa, a very high prevalence, 94.9% (93/98) of resistant *Salmonella* on chicken carcasses purchased from the informal market in South Africa. Of the 170 *Salmonella* isolates recovered from carcass swabs, cloacal swabs and carcass drips, all (100%) exhibited resistance to antimicrobial agents. This study strategy was used because of the unique situation in ‘wet markets’, such as the informal market outlets in Gauteng province which have limited physical infrastructure, lack a potable water supply, have poor drainage, and inadequate wastewater and solid waste disposal [25]. Most importantly, as reported earlier [25], slaughtered chickens are rinsed in infrequently changed water in drums and buckets. The implication is that there is a high potential for cross-contamination among several carcasses with resistant *Salmonella* during the rinsing process. Considering that chickens in the informal market are bagged fresh in nylon bags for sale, the rationale of the study was therefore to collect three types of samples (carcass swabs, cloacal swabs and carcass drips) from each bag. This approach allowed a comparison of the serovars and resistance patterns of *Salmonella* from the three sources to provide evidence of cross contamination. Additionally, the cloacal swab samples were likely to be indicative of the prevalence and resistance patterns from the farms from where the live chickens originated. It cannot be over-emphasized that the high prevalence of resistant *Salmonella* in the chickens sampled, poses a risk of infection or clinical salmonellosis in consumers of improperly cooked *Salmonella*-contaminated chickens. There are also potential therapeutic and public health implications for workers and implications for workers at these outlets.

Overall, across the six townships, the prevalence of resistant *Salmonella* was particularly high to erythromycin (99.6%), spectinomycin (92.6%), streptomycin (82.5%), oxytetracycline (78.0%), and doxycycline (76.1%), which are commonly used by poultry farmers (commercial and backyard) and veterinarians in the country. The detected high prevalence of resistant *Salmonella* in our study has implications for the poultry farms in Gauteng province because all the chickens (broilers, culled breeders and spent hens) processed at the informal market outlets originated from these farms. The overall high prevalence of resistant *Salmonella* may be indicative of improper use or misuse of antimicrobial agents for prophylaxis, growth promotion and therapy which may be as a result of noncompliance by farmers or failure by the authorities to enforce existing legislations [9,10,11,12,13]. The outcome of such a practice is an increase in the prevalence of resistance among pathogens, including *Salmonella*, and therefore resultant therapeutic failure. High prevalence of resistance to antimicrobial agents has been documented for *Salmonella* and other pathogens in the livestock industry in South Africa [17,18,19,33], and in other countries [34]. Additionally, infection of poultry with resistant *Salmonella* on farms has the potential to cause high morbidity and mortality in chickens as earlier reported [1,2,3,4,35]. Furthermore, the potential zoonotic spread of resistant *Salmonella* to workers has been reported [36,37,38].

The high prevalence of resistance to the antimicrobial agents such as erythromycin, spectinomycin, streptomycin, tetracycline, and doxycycline by *Salmonella* in our study agrees with published reports of poultry-associated *Salmonella* by others [39,40,41]. It is pertinent to mention that in South Africa, the tetracyclines are the most commonly used or over-used antibiotics in the livestock industry, particularly in poultry, to treat bacterial infections such as *Salmonella* spp. and *Escherichia coli*, among others [15,16]. This is due to the fact that they are comparatively inexpensive, readily available as over-the-counter veterinary drugs [13,18], and their use is allowed by the South Africa Fertilizers, Farm Feed, Agricultural and Stock Remedies Act (Act 36, 1947). The high prevalence of resistance to tetracycline and doxycycline among *Salmonella* detected in our study is therefore not unexpected. The prevalence of resistance to six antimicrobial agents (tetracycline, doxycycline, streptomycin, spectinomycin, and sulfamethoxazole-trimethoprim) found in *Salmonella* on chickens sampled and processed at the outlets in our study varied significantly across the townships as a result of the origin of the live chickens. This may be due, in part, to the variable use or abuse of the antimicrobial agents on the farms from where the chickens were sourced. This aspect did not form part of our study. The lack of compliance with legislation governing the type and number of antimicrobial agents used in the livestock and human medicine in South African may be a causative factor in the development of antimicrobial resistance of the *Salmonella* isolates studied [17,18,42], as similarly reported by others elsewhere [43,44]. Another potential contributing factor to the significantly different prevalence of resistance to antimicrobial agents among *Salmonella* across townships is the practice of contractors purchasing live chickens from commercial broiler and layer farms across the province and supplying them to the outlets of the informal market [22,28]. Geographical location and farm size are some of the factors that have been reported to significantly facilitate the carriage and spread of *Salmonella* [45,46,47,48].

The variable prevalence of resistant *Salmonella* on these farms will therefore be reflected in our findings at the outlets. This explanation is further supported by the finding of similarly high prevalence (100.0%) of resistant *Salmonella* in the cloacal swab samples which also varied significantly across the townships and measured the carriage of the pathogen in the flocks from where they originated. This is because cloacal swabs of chickens were not subjected to potential cross-contamination experienced by carcass swabs and carcass drips during processing at the outlets. 

Similarly, high prevalence of resistant *Salmonella* has been reported for chickens slaughtered in the outlets of the ‘wet market’ in other countries, such as in the cecal contents of chickens slaughtered in ‘pluck shops’ in Trinidad [46] where 100.0% of the *Salmonella* isolates were resistant to antimicrobial agents which included 10 of the 16 used in the current study and in India where 100.0% (51/51) of *Salmonella* isolates from the rectal swabs in poultry exhibited resistance to 16 antimicrobial agents [49]. 

Equally of potential therapeutic importance was the detection of a very high (94.0%) prevalence of MDR isolates of *Salmonella.* Other studies of the pathogens of poultry in South Africa have similarly reported a high prevalence of MDR in *Salmonella* isolates in chickens and other livestock, thus highlighting their therapeutic and public health implications [42,50,51,52,53,54,55,56]. The prevalence of MDR in the current study is higher than was reported, also using the disc diffusion method, for *Salmonella* from chickens (caeca) in Trinidad, 14.3% [46], Portugal, 75.0% [57], China, 80.0% [58], and in the USA, 92.0% [59], but lower than that reported for isolates in Nepal, 100% [60], Mauritius, 100% [61] and Trinidad, 100.0% [41] and globally [34]. 

It is of public health significance that all (100.0%) the 54 isolates of *Salmonella* from carcass swabs exhibited resistance to one or more antimicrobial agent, and more importantly that 72.2% of these were MDR isolates, thereby posing a potential for zoonotic spread of resistant *Salmonella* and a possible risk of therapeutic failure in workers at the informal market. There are reports of infections by *Salmonella* and other zoonoses associated with exposure of workers on farms and during processing of slaughtered livestock [36,37,38]. This is important considering that workers at the unregulated, illegal outlets of the informal market have been reported to practice poor hygiene in unsanitary environments [22,23], thus contributing to the cross-contamination of carcasses and exposure of the workers to the pathogen at these outlets. The possible exposure of consumers to improperly cooked chickens that are contaminated with resistant *Salmonella* is a food safety concern that can lead to salmonellosis with therapeutic implications, as documented by others [6,7,8]. 

The prevalence of resistance to six antimicrobial agents (tetracycline, doxycycline, streptomycin, spectinomycin, and sulfamethoxazole-trimethoprim) of *Salmonella* on chickens sampled and processed at the outlets in our study varied significantly across the townships as a result of the origin of live chickens. This may be due, in part, to the variable use or abuse of the antimicrobial agents on the farms from where the chickens are sourced. This aspect did not form part of our study. The lack of compliance with legislation governing the type and number of antimicrobial agents used in the livestock and human medicine in South African may be a causative factor in the development of antimicrobial resistance of *Salmonella* isolates studied [17,18,42], as similarly reported by others elsewhere [43,44]. 

It was of interest to have detected that the resistance pattern (E-OXT-S-SPE-DO) was predominant among isolates from the three types of samples and was detected at frequencies of 42.6%, 41.1% and 43.3% among carcass swabs, cloacal swabs and carcass drips, respectively. The differences were found not to be statistically significantly, which may be suggestive of cross-contamination of resistant *Salmonella* among the three types of samples. However, among the isolates which exhibited MDR, the frequency was statistically significantly (*p* = 0.0308) lower for carcass swabs (72.2%) compared with cloacal swabs (92.8%) and carcass drip (80.0%). These findings suggest that cross contamination may not be the only factor involved and therefore follow-up investigation may be required. 

In our study the prevalence of antimicrobial resistant *Salmonella* in carcass drips was generally higher to antimicrobial agents compared to isolates from carcass swabs and cloacal swabs. It has also been reported that the prevalence of *Salmonella* was significantly higher than that found in either the carcass or cloacal swabs of the same chickens sampled from the informal market in Gauteng province [32]. 

A considerable variation of the prevalence of resistant *Salmonella* was detected within and between the nine serovars identified in the current study. Of relevance is that statistically significant differences were detected in the prevalence of resistance to tetracycline, doxycycline, streptomycin, and gentamycin among the serovars of *Salmonella*. This may reflect the differences in the antimicrobial resistance of *Salmonella* serotypes and the frequency of use or abuse of antimicrobial agents across the farms from where the chickens originated. Our findings agree with the reports of other studies conducted in Trinidad [41,46], Spain [62], Iran [63], USA [64], Korea [65], and Malaysia [66], where different prevalence of resistant *Salmonella* was detected among the serovars isolated.

## 4. Conclusions

Our findings of a high prevalence of resistance to antimicrobial agents and MDR isolates of *Salmonella* in chickens processed and retailed at outlets of the informal market in Gauteng province are likely to be indicative of potential zoonotic, food safety and therapeutic implications posed to workers at the outlets and consumers of improperly cooked *Salmonella*-contaminated chicken meat. The findings also indicate a high prevalence of resistant *Salmonella* on commercial poultry farms from where most of the slaughtered chicken originated in Gauteng province, South Africa. It is therefore imperative for workers at the informal market to practice good sanitation so as to prevent or reduce their exposure to resistant *Salmonella.* More importantly, it is prudent to enforce existing regulations on the use of antimicrobial agents on poultry farms in Gauteng province and in South Africa at large, in order to reduce the occurrence of antimicrobial resistant strains of *Salmonella* and other pathogens.

## 5. Materials and Methods

### 5.1. Study Design

The study was designed to determine the antimicrobial profiles of *Salmonella* strains isolated from chicken swabs, cloacal swabs and carcass drips collected from outlets of the informal market in Gauteng province, South Africa in an earlier study (32). The earlier study focused primarily on the prevalence, serovars and factors associated with *Salmonella* contamination of chicken carcasses at these outlets. The current study was designed to relate the prevalence of resistance to antimicrobial agents among *Salmonella* strains to the townships where the outlets sampled were located, the types of samples collected and processed and the serovars of the isolates. This study used a strategy to avoid duplication of isolates of *Salmonella* caused by the criteria employed to select and test isolates in the previous study [32].

### 5.2. Selection of Informal Market Outlets for the Study

To select outlets of the informal market for the study, different categories of outlets identified during the preliminary visits were used. The criteria used for the number of samples collected from each outlet and township, and from each selected outlet were described earlier [22,25]. The types of chickens sampled at the outlets included culled breeders, spent chickens, and broilers which originated from both large commercial and Developing Poultry Farmers Organization (DPFO), as well as from backyard farms in Gauteng province.

### 5.3. Isolation, Identification and Confirmation of Salmonella

The protocol used for the isolation and identification of *Salmonella* from chicken swabs, cloacal swabs and carcass drips was earlier described [14,25,32]. Briefly, each of the 151 chicken samples were inoculated into two enrichment broths, Rappaport Vassiliadis Soya (RVS) broth and Mueller–Kauffman tetrathionate (MKTT) broth and incubated for 24 h at 41.5 and 37 °C, respectively. Thereafter, the growths in the broths were inoculated and streaked for isolation on two selective media, xylose-lysine-desoxycholate (XLD) agar and Brilliance Salmonella agar (BSA) plates which were incubated aerobically at 37 °C for 24 h. Overall, each sample was therefore in effect plated on four agar media combinations of RVS/BSA, RVS/XLD, MKTT/BSA, and MKTT/XLD. Thereafter, isolates that exhibited phenotypic characteristic of *Salmonella* on BSA and XLD were subjected to biochemical tests to presumptively identify the organism [55]. *Salmonella* Typhimurium American Type Culture Collection (ATCC) 14028, was used as the positive control. The slide agglutination test, using *Salmonella* polyvalent O antisera (A-1 & Vi) and a commercial test kit (Thermo Fisher, South Africa) was used to tentatively confirm the presumptive *Salmonella* isolates.

To confirm all tentatively identified *Salmonella*, DNA was extracted from each isolate by the boiling method [16,67] followed by the use of conventional polymerase chain reaction (PCR) to detect the invA gene as described by Rodriguez-Lazaro et al. [67]. The following primer sequences were used to amplify a 284 bp fragment of the invA gene, Forward: 5′ GTGAAATTATCGCCACGTTCGGGCAA 3′ and Reverse: 5′ TCATCGCACCGTCAAAGGAACC 3′ as described by Oliviera et al. [68].

### 5.4. Determination of Serovars of Salmonella by PCR and Conventional Slide Agglutination Test 

The serovars of the isolates of *Salmonella* were determined using conventional PCR as described by Kim et al. [69]. The conventional PCR consisted of two five-plex and two monoplex reactions [69]. The monoplex was used to differentiate between serovars Dublin and Enteritidis with the PT4 primer, and serovars Anatum and Saintpaul were distinguished with the use of STM7 primer set. Table 5 shows the primer sets used in the study. For the study, *Salmonella* isolates that were typable by PCR were randomly selected from the nine serovars and subjected to the conventional agglutination test. The results were interpreted using the Kauffman–White scheme [70] at the Bacteriology Laboratory of the Onderstepoort Veterinary Research, South Africa.

### 5.5. Criteria for Selecting Isolates of Salmonella 

From all the 186 isolates of *Salmonella* recovered from the selective agar (RVS/BSA, RVS/XLD, MKTT/BSA, MKTT/XLD) and confirmed by PCR [32], to reduce the potential for duplicate isolates from each of the four types of samples, the following criteria were used for selection: (a) if a sample yielded several isolates that belonged to the same serovar, one isolate was randomly selected. (b) If a sample yielded isolates that belonged to more than one serovar, one isolate representing each serovar was selected. (c) If several isolates from the same sample were determined to be nontypable by PCR, one isolate was randomly selected.

Using the aforementioned criteria, a total of 170 isolates of *Salmonella* were selected for the study to determine their antimicrobial profiles. With the application of the criteria (a–c) and considering a bagged chicken as a unit from where three types of samples (carcass swabs, cloacal swabs and carcass drips) were collected, the frequency of resistance to antimicrobial agents in chicken-related *Salmonella* was determined (Table 1), i.e., chicken-specific frequency of resistance. The frequency of resistance to antimicrobial agents in 170 isolates of *Salmonella* according to the type of samples tested was also determined (Table 2), i.e., sample type specific frequency of resistance.

### 5.6. Selection of Antimicrobial Agents Used in the Study

Based on consultation with veterinarians in Gauteng province regarding the antimicrobial agents generally used in the livestock industry, including in poultry production, antimicrobial agents were selected for the study. Chloramphenicol was included for comparison and research purposes only since its use in food animals has been banned globally. A total of 16 antimicrobial agents belonging to eight classes of antimicrobial agents (Table 5) were selected for this study.

### 5.7. Determination of Resistance of Salmonella Isolates to Antimicrobial Agents

To determine the antimicrobial resistance profiles of *Salmonella* isolated from chickens sampled at the informal market outlets, the disc diffusion method was used according to the guidelines and interpretation of Clinical and Laboratory Standards Institute, CLSI [71]. The 16 antimicrobial agents (Thermo-Fisher Scientific, Hampshire, United Kingdom), the antimicrobial classes and their concentrations are shown in Table 6. Serotype Typhimurium ATCC 14028 was used as a reference strain.

### 5.8. Statistical Analysis

The data were analyzed using Statistical Package for Social Science (version 25, IBM) to determine whether there were statistically significant differences in the frequency of isolation of antimicrobial resistant *Salmonella* (the independent variable), and dependent variables, specifically, the location of outlets in six townships, the types of samples processed (carcass swabs, cloacal swabs and carcass drips), and the serovars of the isolates. Data were presented as frequencies (percentages) and any association determined using chi square and Fisher’s exact tests with the level of significance set at an alpha level of 0.05. 

## Figures and Tables

**Table 1:** Prevalence of resistant *Salmonella* in chickens sampled from the informal market in Gauteng province, South Africa.

	No. (%) of Resistant *Salmonella* Isolates from Chickens ^a^ Sampled from Townships:							
	Atteridgeville	Garanguwa	Tembisa/Modise	Alexandra	Germiston	Soweto		Total
Antimicrobial Agent	(*n* = 4) ^b^	(*n* = 5)	(*n* = 1)	(*n* = 13)	(*n* = 20)	(*n* = 55)	*p*-Value	(*n* = 98)
Erythromycin (E)	4 (100.0)	3 (60.0)	1 (100.0)	10 (76.9)	20 (100.0)	55 (100.0)	<0.001	93 (94.9)
Oxytetracycline (OXT)	2 (50.0)	0 (0.0)	0 (0.0)	5 (38.5)	11 (55.0)	46 (83.6)	<0.001	64 (65.3)
Chloramphenicol (C)	0 (0.0)	0 (0.0)	0 (0.0)	0 (0.0)	0 (0.0)	2 (3.6)	0.902	2 (2.0)
Kanamycin (K)	1 (25.0)	0 (0.0)	0 (0.0)	0 (0.0)	0 (0.0)	1 (1.8)	0.044	2 (2.0)
Nalidixic acid (NA)	0 (0.0)	0 (0.0)	0 (0.0)	0 (0.0)	0 (0.0)	1 (1.8)	0.978	1 (1.0)
Streptomycin (S)	3 (75.0)	0 (0.0)	0 (0.0)	6 (46.2)	15 (75.0)	49 (89.1)	<0.001	73 (74.5)
Spectinomycin (SPE)	4 (100.0)	0 (0.0)	0 (0.0)	7 (53.8)	19 (95.0)	51 (87.9)	<0.001	81 (82.7)
Ciprofloxacin (CIP)	0 (0.0)	0 (0.0)	0 (0.0)	0 (0.0)	1 (5.0)	0 (0.0)	0.558	1 (1.0)
Ampicillin (AMP)	0 (0.0)	0 (0.0)	0 (0.0)	0 (0.0)	2 (10.0)	3 (5.5)	0.815	5 (5.1)
Cefotaxime (CET)	1 (25.0)	0 (0.0)	0 (0.0)	1 (7.7)	6 (30.0)	7 (12.7)	0.351	15 (15.3)
Doxycycline (DO)	2 (50.0)	0 (0.0)	0 (0.0)	4 (30.8)	10 (50.0)	42 (76.4)	0.001	58 (59.2)
Gentamycin (CN)	0 (0.0)	0 (0.0)	0 (0.0)	0 (0.0)	2 (10.0)	0 (0.0)	0.158	2 (2.0)
Amoxicillin-clavulanic acid (AMOX)	0 (0.0)	0 (0.0)	0 (0.0)	1 (7.7)	1 (5.0)	2 (3.6)	0.968	4 (4.1)
Sulfamethoxazole-trimethoprim (SXT)	2 (50.0)	0 (0.0)	0 (0.0)	4 (30.8)	1 (5.0)	2 (3.6)	0.003	9 (9.2)
Ceftazidime (CAZ)	0 (0.0)	0 (0.0)	0 (0.0)	0 (0.0)	2 (10.0)	1 (1.8)	0.513	3 (3.1)
Norfloxacin (NOR)	0 (0.0)	0 (0.0)	0 (0.0)	1 (7.7)	0 (0.0)	0 (0.0)	0.252	1 (1.0)
*p*-value	<0.001	<0.001	0.382	<0.001	<0.001	<0.001		

^a^ Isolates of *Salmonella* originally recovered from 151 chickens distributed across six townships comprising Atteridgeville (23), Garanguwa (18), Tembisa/Modise (10), Alexandra (20), Germiston (20), and Soweto (60), respectively as reported earlier by Mokgophi et al. [32]. ^b^ For a total of 98 *Salmonella*-positive chickens, the chicken-specific frequency of antimicrobial resistant *Salmonella* recovered was determined.

**Table 2:** Prevalence of antimicrobial resistance in *Salmonella* isolates by type of sample.

	No. (%) of Resistant *Salmonella* Isolates by Type of Sample Collected:			
	Carcass Swab	Cloacal Swab	Carcass Drip	*p*-Value
Antimicrobial Agent	(*n* = 54) ^a^	(*n* = 56) ^a^	(*n* = 60) ^a^	
Erythromycin (E)	54 (100.0)	56 (100.0)	60 (100.0)	NA
Oxytetracycline (OXT)	37 (68.5)	44 (78.6)	46 (76.7)	0.436
Chloramphenicol (C)	0 (0.0)	0 (0.0)	2 (3.3)	0.156
Kanamycin (K)	0 (0.0)	4 (7.1)	1 (1.7)	0.066
Nalidixic acid (NA)	0 (0.0)	1 (1.8)	1 (1.7)	0.623
Streptomycin (S)	34 (63.0)	48 (85.7)	54 (90.0)	<0.001
Spectinomycin (SPE)	46 (85.2)	51 (91.1)	54 (90.0)	0.580
Ciprofloxacin (CIP)	0 (0.0)	1 (1.8)	0 (0.0)	0.359
Ampicillin (AMP)	0 (0.0)	5 (8.9)	3 (5.0)	0.086
Cefotaxime (CET)	2 (3.7)	13 (23.2)	9 (15.0)	0.0130
Doxycycline (DO)	35 (64.8)	12 (21.4)	42 (70.0)	<0.001
Gentamycin (CN)	0 (0.0)	3 (5.4)	3 (5.0)	0.234
Amoxicillin-clavulanic acid (AMOX)	2 (3.7)	4 (7.1)	1 (1.7)	0.327
Sulfamethoxazole-trimethoprim (SXT)	0 (0.0)	5 (8.9)	6 (10.0)	0.063
Ceftazidime (CAZ)	1 (1.9)	4 (7.1)	1 (1.7)	0.201
Norfloxacin (NOR)	0 (0.0)	1 (1.8)	1 (1.7)	0.623
*p*-value	<0.001	<0.001	<0.001	

^a^ A total of 170 isolates of *Salmonella* recovered from carcass swabs (*n* = 54), cloacal swabs (*n* = 56), and carcass drips (*n* = 60), based on their serovars were tested for their resistance to antimicrobial agents to determine sample type-specific frequency of resistance; NA: Not applicable.

**Table 3:** Frequency of resistant *Salmonella* isolates belonging to different serovars isolated from chickens in the informal market in Gauteng province, South Africa.

	No. (%) of *Salmonella* Isolates Belonging to Nine Serotypes Resistant to Antimicrobial Agents:										
	Bovismorbificans	Hadar	Dublin	Enteritidis	Mbandaka	Saintpaul	Thompson	Infantis	Agona		Total
Antimicrobial Agents	(*n* = 41) ^a^	(*n* = 18)	(*n* = 13)	(*n* = 11)	(*n* = 9)	(*n* = 6)	(*n* = 2)	(*n* = 2)	(*n* = 1)	*p*-Value	(*n* = 103)
Erythromycin	41 (100.0)	18 (100.0)	13 (100.0)	11 (100.0)	9 (100.0)	6 (100.0)	2 (100.0)	2 (100.0)	1 (100.0)	NA	103 (100.0)
Oxytetracycline	38 (92.7)	11 (61.1)	10 (76.9)	9 (81.8)	7 (77.8)	4 (66.7)	1 (50.0)	1 (50.0)	1 (100.0)	0.188	84 (81.6)
Chloramphenicol	0 (0.0)	1 (5.6)	0 (0.0)	1 (9.1)	0 (0.0)	0 (0.0)	0 (0.0)	0 (0.0)	0 (0.0)	0.686	2 (1.9)
Kanamycin	1 (2.4)	1 (5.6)	0 (0.0)	0 (0.0)	0 (0.0)	0 (0.0)	0 (0.0)	0 (0.0)	0 (0.0)	0.976	2 (1.9)
Nalidixic acid	0 (0.0)	1 (5.6)	0 (0.0)	0 (0.0)	0 (0.0)	0 (0.0)	0 (0.0)	0 (0.0)	0 (0.0)	0.782	1 (1.0)
Streptomycin	38 (92.7)	15 (83.3)	11 (84.6)	9 (81.8)	8 (88.9)	2 (33.3)	1 (50.0)	1 (50.0)	1 (100.0)	0.029	83.5
Spectinomycin	39 (95.1)	16 (88.9)	12 (92.3)	9 (81.8)	7 (77.8)	6 (100.0)	1 (50.0)	1 (50.0)	1 (100.0)	0.213	92 (89.3)
Ciprofloxacin	0 (0.0)	0 (0.0)	0 (0.0)	0 (0.0)	0 (0.0)	0 (0.0)	0 (0.0)	0 (0.0)	0 (0.0)	NA	0
Ampicillin	0 (0.0)	0 (0.0)	0 (0.0)	1 (9.1)	0 (0.0)	0 (0.0)	0 (0.0)	0 (0.0)	0 (0.0)	0.391	1 (1.0)
Cefotaxime	1 (2.4)	3 (16.7)	3 (23.1)	2 (18.2)	2 (22.2)	0 (0.0)	1 (50.0)	0 (0.0)	0 (0.0)	0.205	12(11.7)
Doxycycline	34 (82.9)	13 (7	9 (69.2)	9 (81.8)	5 (55.6)	4 (66.7)	0 (0.0)	1 (50.0)	1 (100.0)	0.227	76 (73.8)
Gentamycin	2 (4.9)	0 (0.0)	0 (0.0)	0 (0.0)	0 (0.0)	0 (0.0)	0 (0.0)	0 (0.0)	0 (0.0)	0.929	2 (1.9)
AmoxycillinClavulanic acid	3 (7.3)	0 (0.0)	0 (0.0)	2 (18.2)	0 (0.0)	0 (0.0)	0 (0.0)	0 (0.0)	0 (0.0)	0.497	5 (4.9)
Sulfamethoxazole-trimethoprim	2 (4.9)	1 (5.6)	0 (0.0)	1 (9.1)	1 (11.1)	0 (0.0)	0 (0.0)	0 (0.0)	0 (0.0)	0.965	5 (4.9)
Ceftazidime	1 (2.4)	0 (0.0)	0 (0.0)	1 (9.1)	0 (0.0)	0 (0.0)	0 (0.0)	0 (0.0)	0 (0.0)	0.856	2 (1.9)
Norfloxacin	1 (2.4)	0 (0.0)	0 (0.0)	0 (0.0)	0 (0.0)	0 (0.0)	0 (0.0)	0 (0.0)	0 (0.0)	0.992	1 (1.0)
*p*-value	<0.001	<0.001	<0.001	<0.001	<0.001	<0.001	0.220	0.220	0.382		

^a^ Among all the 41 isolates that belonged to serovar Bovismorbificans, resistance was exhibited to 12 different antimicrobial agents tested (i.e., 75.0%, 12/16) by at least one isolate.

**Table 4:** Resistance patterns exhibited by *Salmonella* isolates recovered by types of samples collected.

	No. (%) of Isolates of *Salmonella* with Resistance Patterns from:			
	No. of Antimicrobial	Carcass Swabs	Cloacal Swabs	Carcass Drips
Resistance Pattern ^a^	Agents in Pattern	(*n* = 54)	(*n* = 56)	(*n* = 60)
E-OXT-S-SPE-DO	5	23 (42.6)	23 (41.1)	26 (43.3)
E-SPE	2	6 (11.1)	0 (0.0)	1 (1.7)
E-OXT-SPE-DO	4	6 (11.1)	0 (0.0)	0 (0.0)
E	1	6 (11.1)	2 (3.6)	6 (10.0)
E-OXT-S-SPE-D0-AMOX	6	2 (3.7)	0 (0.0)	0 (0.0)
E-S-SPE-DO	4	2 (3.7)	1 (1.8)	0 (0.0)
E-OXT-S-SPE	4	2 (3.7)	0 (0.0)	0 (0.0)
E-OXT-S-SPE-CET-DO	6	0 (0.0)	5 (8.9)	3 (5.0)
E-OXT-S-DO	4	0 (0.0)	3 (5.4)	1 (1.7)
E-SPE-CET	3	0 (0.0)	2 (3.6)	0 (0.0)
E-S-SPE-CET	4	1 (1.9)	2 (3.6)	0 (0.0)
E-S-SPE	3	2 (3.7)	2 (3.6)	3 (5.0)
E-OXT-S-SPE-DO-CN	6	0 (0.0)	2 (3.6)	0 (0.0)
E-OXT-K-S-SPE-DO	6	0 (0.0)	2 (3.6)	0 (0.0)
E-OXT-S-SPE	4	0 (0.0)	0 (0.0)	3 (5.0)
E-OXT-S-SPE-DO-SXT-NOR	7	0 (0.0)	0 (0.0)	2 (3.3)
E-0XT-S-SPE-DO-SXT	6	0 (0.0)	1 (1.8)	2 (3.3)
E-SPE-DO	3	1 (1.9)	0 (0.0)	1 (1.7)
E-OXT-S	3	1 (1.9)	0 (0.0)	0 (0.0)
E-OXT-DO	3	1 (1.9)	0 (0.0)	1 (1.7)
E-OXT-S-SPE-CET-DO-CEFT	7	1 (1.9)	0 (0.0)	0 (0.0)
E-OXT-S-SPE-CET-D0-SXT	7	0 (0.0)	1 (1.8)	0 (0.0)
E-OXT-K-S-SPE-DO-SXT	7	0 (0.0)	1 (1.8)	0 (0.0)
E-OXT-K-S-SPE-AMP-DO-SXT	8	0 (0.0)	1 (1.8)	0 (0.0)
E-SPE-CET-AMOX-CEFT	5	0 (0.0)	2 (3.6)	0 (0.0)
E-SPE-AMP-CET-CN-CEFT	6	0 (0.0)	1 (1.8)	0 (0.0)
E-OXT-S-SPE-AMP-DO-AMOX-CEFT	8	0 (0.0)	1 (1.8)	0 (0.0)
E-OXT-S-SPE-AMP-CET-DO-AMOX-CEFT	9	0 (0.0)	1 (1.8)	0 (0.0)
E-OXT-S-SPE-DO-SXT	6	0 (0.0)	1 (1.8)	0 (0.0)
E-OXT-S-SPE-AMP-DO-AMOX	7	0 (0.0)	1 (1.8)	0 (0.0)
E-OXT-K-S-SPE-DO	6	0 (0.0)	1 (1.8)	0 (0.0)
E-OXT-S-SPE-DO-AMOX-SXT	7	0 (0.0)	0 (0.0)	1 (1.7)
E-S	2	0 (0.0)	0 (0.0)	1 (1.7)
E-S-SPE-AMP-CET-CN-CEFT	7	0 (0.0)	0 (0.0)	1 (1.7)
E-S-SPE-AMP-CET-CN	6	0 (0.0)	0 (0.0)	1 (1.7)
E-S-SPE-CET-DO	5	0 (0.0)	0 (0.0)	1 (1.7)
E-OXT-S-SPE-SXT	5	0 (0.0)	0 (0.0)	1 (1.7)
E-OXT-C--S-SPE-DO	6	0 (0.0)	0 (0.0)	1 (1.7)
E-OXT-S-SPE-DO-CN	6	0 (0.0)	0 (0.0)	1 (1.7)
E-OXT-C-K-S-SPE-CET-DO-SXT	10	0 (0.0)	0 (0.0)	1 (1.7)

^a^ E—erythromycin, OXT—tetracycline, C—chloramphenicol, K—kanamycin, NA—nalidixic acid, S—streptomycin, SPE—spectinomycin, CIP—ciprofloxacin, AMP—ampicillin, CET—cefotaxime, DO—doxycycline, CN—gentamycin, AMOX—amoxicillin-clavulanic acid, SXT—sulfamethoxazole-trimethoprim, CAZ—ceftazidime, NOR—norfloxacin.

**Table 5:** Primers used for molecular serotyping [70].

Reaction 1 (STM) (Five-Plex)		Product Size (bp)
**STM716F** 5′ ACCGCTGCTTAATCCTGATGG 3′	**STM0716R** 5′ TGGCCCTGAGCCAGCTTTT 3′	187
**STM1350R** 5′ TCAAAATTACCGGGCGCA 3′	**STM1350R** 5′ TTTTAAGACTACATACGCGCATGAA 3′	171
**STM0839F** 5′ TCCAGTATGAAACAGGCAACGTGT 3′	**STM0839R** 5′ GCGACGCATTGTTCGATTGAT 3′	137
**STM4525F** 5′ TGGCGGCAGAAGCGATG 3′	**STM4525R** 5′ CTTCATTCAGCAACTGACGCTGAG 3′	114
**STM4538F** 5′ TGGTCACCGCGCGTGAT 3′	**STM4538R** 5′ CGAACGCCAGGTTCATTTGT 3′	93
**Reaction 2 (STY) (five-plex)**		
**STY0311F** 5′ TGGTATGGTTAAGCGGAGAATGG 3′	**STY0312R** 5′ GAGAGTCATAGCCCACACCAAAG 3′	301
**STY0346F** 5′ GGCTGGAGCAGCCTTACAAAA 3′	**STY0347R** 5′ AAGAGTTGCCTGGCTGGTAAAA 3′	262
**STY2299F** 5′ AATCCCCCCCCCTCAAAAA 3′	**STY2300R** 5′ GGTACACCGTTTACTGTTTGCTGGA 3′	220
**STM3845F** 5′ ATATCTCATCGTCTCCTTTTCGTGT 3′	**STM3845R** 5′ GAAGGTCCGGATAGGCATTCT 3′	181
**STY2349F** 5′ AATTACGGAGCAGCAGATCGAGG 3′	**STY2349R** 5′ TGCGGCCAGCTGTTCAAA 3′	124
**Reaction 3 (PT4) (monoplex)**		
**PT4F** 5′ GGCGATATATAAGTACGACCATCATGG 3′	**PT4R** 5′ GCACGCGGCACAGTTAAAA 3′	225
**Reaction 4 (STM7) (monoplex)**		
**STM2150F** 5′ CATAACCCGCCTCGACCTCAT 3′	**STM2150R** 5′ AGATGTCGTGAGAAGCGGTGG 3′	101

**Table 6:** Classes and concentrations of antimicrobial agents used in the study.

Class of Antimicrobial Agents	Type of Antimicrobial Agents Used	Concentration (µg)
Aminoglycosides	Spectinomycin (SPE)	100
	Streptomycin (S)	10
	Kanamycin (K)	30
	Gentamycin (CN)	10
Beta lactams	Ampicillin (AMP)	10
	Amoxicillin-clavulanic acid (AMOX)	30
Cephalosporin	Ceftazidime (CAZ)	30
	Cefotaxime (CET)	30
Fluoroquinolones	Nalidixic acid (NA)	30
	Norfloxacin (NOR)	10
	Ciprofloxacin (CIP)	5
Macrolides	Erythromycin (E)	15
Phenicols	Chloramphenicol (C)	30
Sulphonamides	Sulfamethoxazole-trimethoprim (SXT)	23.75/1.25
Tetracyclines	Oxytetracycline (OXT)	30
	Doxycycline (DO)	30

## Data Availability

Data available in a publicly accessible repository. The data presented in this study are openly available in UPspace (https://repository.up.ac.za/ (accessed on 27 February 2021) and in FigShare at https://research.up.ac.za/ (accessed on 27 February 2021).
